# Uterine Arteriovenous Malformation Treated with Selective Embolisation of Uterine Arteries: A Case Report

**DOI:** 10.15388/Amed.2021.28.1.17

**Published:** 2021-04-29

**Authors:** Vilius Rudaitis, Gailė Maldutytė

**Affiliations:** Clinic of Obstetrics and Gynaecology, Institute of Clinical Medicine, Faculty of Medicine, Vilnius University, Vilnius, Lithuania Department of Gynaecology, Center of Obstetrics and Gynaecology, Vilnius University Hospital Santaros Klinikos, Vilnius, Lithuania; Faculty of Medicine, Vilnius University, Vilnius, Lithuania Department of Gynaecology, Center of Obstetrics and Gynaecology, Vilnius University Hospital Santaros Klinikos, Vilnius, Lithuania

**Keywords:** uterine arteriovenous malformation, uterine vascular lesion, embolisation

## Abstract

Uterine arteriovenous malformation (AVM) is a very rare gynaecological condition, which can potentially lead to life-threatening abnormal uterine bleeding. In most cases uterine AVM is associated with prior pregnancy or pelvic surgery. We present the case of young woman seven weeks after medical termination of pregnancy diagnosed with heavy uterine bleeding due to uterine AVM, which was successfully treated with selective embolisation of uterine arteries.

## Abstract (lt)

**Sėkmingas gimdos arterioveninės malformacijos gydymas, atliekant selektyvią gimdos arterijų embolizaciją: klinikinio atvejo pristatymas:**

**Santrauka.** Gimdos arterioveninė malformacija yra labai reta ir pavojinga patologija, galinti sukelti gausų kraujavimą iš gimdos. Dažniausiai šią būklę lemia ankstesnis nėštumas ar dubens operacija. Mes pristatome klinikinį atvejį apie jauną moterį, kuriai po medikamentinio nėštumo nutraukimo praėjus septynioms savaitėms išsivystė gimdos arterioveninė malformacija, pasireiškusi nenormaliu kraujavimu iš gimdos. Jį pavyko sėkmingai išgydyti atlikus selektyvią gimdos arterijų embolizaciją.

**Raktažodžiai:** gimdos arterioveninė malformacija, gimdos kraujagyslinė patologija, embolizacija

## Introduction

Uterine arteriovenous malformation (AVM) is a vascular pathology, distinguished by its abnormal communication between branches of the uterine artery and the venous plexuses within myometrium [1, 2]. These lesions are classified as congenital and acquired. Although incidence of uterine AVM is only 0.1%, in literature, acquired uterine AVMs are more frequently established [3, 4]. Generally they present in reproductive age women as heavy or irregular vaginal bleeding in the postpartum period or a few weeks after spontaneous miscarriage or termination of pregnancy [1, 4]. The treatment depends on the AVM’s severity of symptoms, desire for future fertility, localisation and size of the lesion. Embolisation of the uterine arteries is safe and effective treatment option, which preserves future fertility [2-5, 8-10].

## Case report

A 34-year-old woman, gravida 2, para 1, abortion 1, was admitted to Vilnius University Hospital Santaros Klinikos Gynaecology department after the episode of sudden and heavy vaginal bleeding. Seven weeks prior to her admission, she underwent an uncomplicated medical termination of pregnancy for foetal anomaly (acrania) at 13 weeks of gestation (dilation and curettage was not performed). Histopathological analysis confirmed previously diagnosed foetal anomaly without additional pathological findings. Three years ago she had a vacuum-assisted vaginal delivery with manual removal of placenta. Both pregnancies were achieved following in vitro fertilization for unknown reason of infertility. Based on her gynaecological history, she had two previous hysteroscopic polypectomies.

**Fig. 1.**
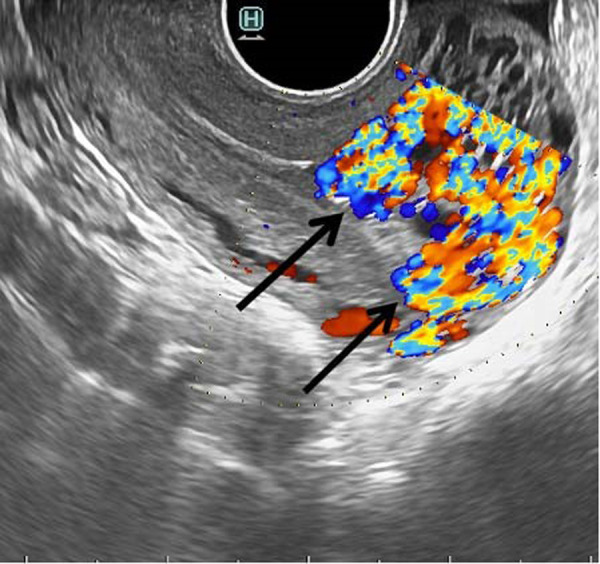
Transvaginal grayscale ultrasound image of the uterus in sagittal plane demonstrates tubular hypoechoic areas within myometrium (white arrow), while colour Doppler shows the lesion of vascular origin (black arrows)

During admission, she was found hemodynamically stable, but moderate vaginal bleeding was observed. Laboratory results were as follows: haemoglobin – 9.5 g/dL, haematocrit – 33.5%, beta-human chorionic gonadotropin – 16.6 U/l (reduced from 31.2 U/l in one week period). Following transvaginal grayscale ultrasonography, multiple hypoechoic tortuous structures within myometrium, involving corpus and fundus of the uterus were revealed and endometrial thickness of 4 mm was noticed. Colour Doppler imaging demonstrated 8.8×5.5 ×5.0 cm vascular lesion within myometrium with multiple arteriovenous shunts (Fig. 1). Spectral Doppler analysis revealed intense vascularity within lesion (peak systolic velocity of 65 cm/s) with multidirectional, low-resistance flow (resistance index of 0.38).

**Fig. 2.**
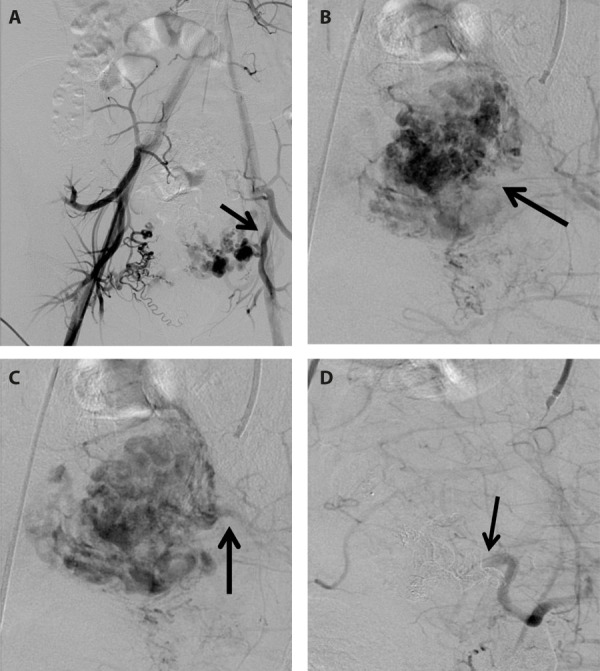
(A) Pelvic angiography demonstrates hypervascular structure arising from the left uterine artery (arrow). (B) Selective angiography of the left internal iliac artery indicates a uterine AVM (arrow). (C) Image of venous phase demonstrates early draining vein (arrow). (D) Post-embolisation angiography reveals total occlusion of the left uterine artery (arrow) and absence of the previous AVM

Clinical findings led to suspicion of uterine AVM. Therefore the patient was referred to interventional radiology department. Pelvic angiography revealed a hypervascular mass supplied mainly by left internal iliac artery, with early venous filling. Selective embolisation of both uterine arteries was performed with ethylene vinyl alcohol copolymer (Onyx, Medtronic) and embolisation gelatin (EmboCube, Merit Medical), while using the right femoral approach. The post-embolisation angiography demonstrated that both uterine arteries had been occluded and resolution of vascular lesion had been achieved (Fig. 2). 

**Fig. 3.**
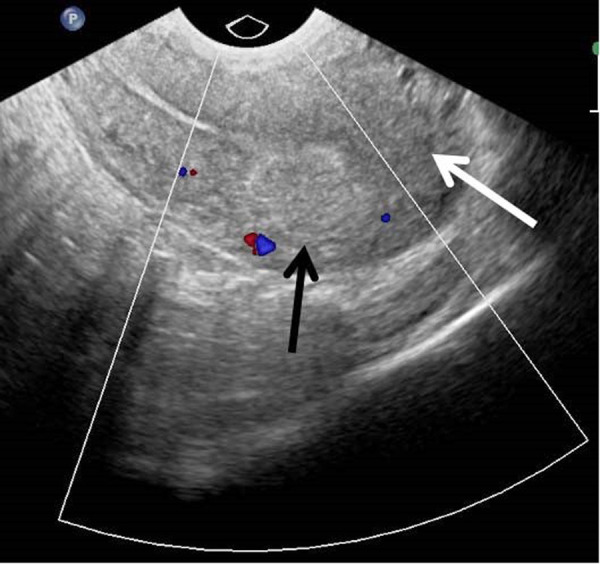
Transvaginal colour Doppler ultrasound image of the uterus in sagittal plane one month after embolisation demonstrates resolution of previous vascular lesion (white arrow), while hyperechoic mass within myometrium (black arrow) indicates embolic agent left post selective embolisation

Following procedure the patient had a mild lower abdominal pain and no other symptoms or complications related to undergone embolisation. The patient was discharged from hospital the next day after embolisation. At one month follow-up visit transvaginal ultrasonography with colour Doppler showed resolution of AVM (Fig. 3) and the patient reported prolonged menstruation already lasting one week. At six month follow-up visit no recurrence of abnormal vaginal bleeding or vascular lesion within myometrium was found.

## Discussion

Uterine AVMs arise from an abnormal communication between branches of the uterine artery and the venous plexuses, forming vascular fistulas within myometrium. Uterine AVMs are classified as congenital and acquired. Congenital uterine AVMs are caused by defect in the differentiation of primitive capillary plexus and typically these lesions extend into the surrounding pelvis [2,4,5]. Acquired AVMs are usually associated with prior pregnancy and may present after dilation and curettage or caesarean section and less frequently after myomectomy, or in the setting of pre-existing pathologic uterine processes [2]. Although uterine AVMs have been reported in both adolescent and postmenopausal women, the mean age of women diagnosed with AVMs is 30 years [5]. 

The most common clinical presentation of uterine AVM is uterine bleeding, which may occur, when the endothelial lining of the vessels in the AVM is disrupted either naturally during menstruation or artificially during curettage [1, 6, 7]. As a result, patients with uterine AVM present with heavy menstrual or intermenstrual bleeding or report persistent postpartum bleeding, abnormal bleeding after abortion or massive bleeding after dilation and curettage [4]. Heavy uterine bleeding may then lead to significant hypotension, anaemia or inevitable loss of uterus. Approximately 50% of patients with uterine AVM require blood transfusion due to secondary anaemia [2, 4, 5]. Some treatment options of uterine bleeding, such as dilatation and curettage may worsen the bleeding, therefore it is important to recognize the uterine AVM and to ensure appropriate treatment [2, 6]. In our case report the patient presented with abnormal uterine bleeding, that occurred seven weeks after medical termination of pregnancy. Although the termination of pregnancy could be the main factor in development of acquired uterine AVM, the manual removal of placenta during previous delivery and multiple hysteroscopic polypectomies could also cause the significant trauma to uterus.

Transvaginal ultrasonography is commonly performed for the initial evaluation of an abnormal uterine bleeding [4]. Sonographic grayscale features of AVMs are nonspecific and constitute of multiple irregular hypoechoic, tortuous and tubular structures within the myometrium [1, 2, 5-7]. Colour Doppler ultrasonography provides a more specific image and presents a hypervascular area within myometrium while containing tortuous vessels with irregular turbulent flow [4, 6, 7]. The low resistance and high systolic velocity flow within the lesion is highly suggestive for uterine AVM [1, 7]. Magnetic resonance imaging and computed tomography are also useful non-invasive methods for further evaluation of uterine AVMs [4]. However, the pelvic angiography is considered the first line approach for diagnosing the uterine AVM. It enables identification of abnormal vascular formations, determines the feeding artery and reveals the early venous filling, which is specific feature for AVM. Because angiography is an invasive procedure, it is performed for treatment purposes only, in cases where selective embolisation of uterine arteries has been considered as the best possible treatment [4, 6]. Sonographic features demonstrated in our case are characteristic to uterine AVM. Retained products of conception and gestational trophoblastic disease may also have similar sonographic features to those of uterine AVM, therefore the differentiation between them is essential [6]. Retained products of conception on grayscale ultrasound images appear as hyper-echoic mass within endometrial cavity and may be suspected, when endometrial thickness is more than 8–10 mm [1]. In our case, the diagnosis of retained products of conception have been ruled out, because there was no mass found within an endometrial cavity and endometrium was only 4 mm thick. Also, this patient did not have high or increasing pattern of beta-human chorionic gonadotropin levels, which is known to be characteristic for gestational trophoblastic disease. Neither retained products of conception, nor gestational trophoblastic disease during pelvic angiography demonstrate early venous filling. However, in our case the early venous filling was noted confirming AVM diagnosis. 

Management of uterine AVM depends on the severity of symptoms, desire for future fertility, localisation and size of the lesion. Historically, symptomatic uterine AVM was treated only by hysterectomy until 1982 [11], then the first embolisation of uterine arteries as a treatment of uterine AVM was reported [2-4, 10]. Nowadays hysterectomy is only performed in patients with recurrent heavy uterine bleeding, in cases when alternative treatment options have failed or the access to medical facilities is limited and remains the most definitive treatment option [2, 4]. Selective embolisation of uterine arteries is considered the first line fertility preserving treatment in symptomatic cases of uterine AVM. Control of uterine bleeding and resolution of uterine AVM is achieved in 88% to 94.1% cases [9, 10]. Asymptomatic patients with uterine AVM or those presenting with light uterine bleeding may be treated conservatively. Medical management of uterine AVM may include methylergonovine maleate, tranexamic acid, danazol, combined oral contraceptive pills, gonadotrophin‐releasing hormone agonists and in long-term has demonstrated having a positive effect on AVM’s regression. Expectant management may be an option for asymptomatic patients with uterine AVM [3, 6]. It is recommended to follow up these patients until full clinical and radiological resolution of uterine AVM [2]. In our case, the resolution of vaginal bleeding and uterine AVM was also achieved by selective embolisation of uterine arteries and at six month follow-up outpatient visit no recurrence was found.

Menstruations in patients following selective embolisation of uterine arteries usually reappear within 1–2 months [5]. Although there is a higher risk of prematurity and intrauterine growth restriction, successful intrauterine pregnancies have been reported after selective embolization of uterine arteries [5, 6, 9, 10]. In such cases normal vaginal delivery should always be recommended, unless caesarean section is indicated by other clinical reason [5]. 

## Conclusions

Uterine AVM is a very rare gynaecological condition. In all reproductive age women presenting with abnormal uterine bleeding in the postpartum period or within a few weeks after spontaneous miscarriage or termination of pregnancy, or after recent pelvic surgery a uterine AVM should be suspected. Selective embolization of uterine arteries is safe, effective and fertility preserving treatment of choice for symptomatic uterine AVM.
